# RNA-seq assistant: machine learning based methods to identify more transcriptional regulated genes

**DOI:** 10.1186/s12864-018-4932-2

**Published:** 2018-07-20

**Authors:** Likai Wang, Yanpeng Xi, Sibum Sung, Hong Qiao

**Affiliations:** 1 Institute for Cellular and Molecular Biology, The University of Texas at Austin, 2506 Speedway, NMS 5.324, Austin, TX 78712 USA; 2 Department of Molecular Biosciences, The University of Texas at Austin, 2506 Speedway, NMS 5.324, Austin, TX 78712 USA

**Keywords:** Machine learning, Differentially expressed genes (DEGs), Ethylene, *Arabidopsis*

## Abstract

**Background:**

Although different quality controls have been applied at different stages of the sample preparation and data analysis to ensure both reproducibility and reliability of RNA-seq results, there are still limitations and bias on the detectability for certain differentially expressed genes (DEGs). Whether the transcriptional dynamics of a gene can be captured accurately depends on experimental design/operation and the following data analysis processes. The workflow of subsequent data processing, such as reads alignment, transcript quantification, normalization, and statistical methods for ultimate identification of DEGs can influence the accuracy and sensitivity of DEGs analysis, producing a certain number of false-positivity or false-negativity. Machine learning (ML) is a multidisciplinary field that employs computer science, artificial intelligence, computational statistics and information theory to construct algorithms that can learn from existing data sets and to make predictions on new data set. ML–based differential network analysis has been applied to predict stress-responsive genes through learning the patterns of 32 expression characteristics of known stress-related genes. In addition, the epigenetic regulation plays critical roles in gene expression, therefore, DNA and histone methylation data has been shown to be powerful for ML-based model for prediction of gene expression in many systems, including lung cancer cells. Therefore, it is promising that ML-based methods could help to identify the DEGs that are not identified by traditional RNA-seq method.

**Results:**

We identified the top 23 most informative features through assessing the performance of three different feature selection algorithms combined with five different classification methods on training and testing data sets. By comprehensive comparison, we found that the model based on InfoGain feature selection and Logistic Regression classification is powerful for DEGs prediction. Moreover, the power and performance of ML-based prediction was validated by the prediction on ethylene regulated gene expression and the following qRT-PCR.

**Conclusions:**

Our study shows that the combination of ML-based method with RNA-seq greatly improves the sensitivity of DEGs identification.

**Electronic supplementary material:**

The online version of this article (10.1186/s12864-018-4932-2) contains supplementary material, which is available to authorized users.

## Background

Differentially expressed genes (DEGs) have been widely used to understand not only gene function but also the molecular mechanisms underlying different biological processes. A number of methods have been developed to analyze differential gene expression, such as Real-time reverse transcription PCR (qRT-PCR) [1], cDNA microarray analysis [2], whole genome tiling array [3–5], RNA sequencing (RNA-seq) [6–8]. As a result of the low cost of next generation sequencing technologies and its remarkable power and accuracy, RNA-seq has become the most popular method for DEGs analysis.

Although different quality controls have been applied at different stages of the sample preparation and data analysis to ensure both reproducibility and reliability of RNA-seq results, there are still limitations or bias on the detectability for certain DEGs [9–11]. Whether the transcriptional dynamics of a gene can be captured accurately depends on experimental design/operation and the following data analysis processes [9, 12, 13]. The workflow of subsequent data processing, such as reads alignment, transcript quantification, normalization, and statistical methods for ultimate identifying of DEGs can influence the accuracy and sensitivity of DEGs analysis, producing a certain number of false-positivity or false-negativity [14–16].

Machine learning (ML) is a multidisciplinary field that employs computer science, artificial intelligence, computational statistics and information theory to construct algorithms that can learn from existing data sets and to make predictions on new data set [17]. It is increasingly a key tool for biological studies, including biological image analysis [18], cancer study [19, 20], robust phenotyping [21], as well as gene discovery [22–24]. ML–based differential network analysis has been applied to predict stress-responsive genes through learning the patterns of 32 expression characteristics of known stress-related genes [23]. In addition, the epigenetic regulation plays critical roles in gene expression, therefore, DNA and histone methylation data has been shown to be powerful for ML-based model for prediction of gene expression in lung cancer [19]. Therefore, ML-based methods are able to assist with the identification of DEGs that are missed by a regular RNA-seq data analysis method.

Take plant response to ethylene as an example: ethylene is a small volatile hydrocarbon gas and regulates a wide variety of developmental processes and stress responses in plant cells. Signal transduction of ethylene has been studied for more than 2 decades, mainly with the model plant *Arabidopsis*, and a linear signal transduction pathway has been proposed [25, 26]. Ethylene regulated genes have been determined using RNA-seq in *Arabidopsis* etiolated seedlings [6, 8, 27, 28], in which many genes have been confirmed to be regulated by ethylene treatment, such as *CONSTITUTIVE TRIPLE RESPONSE 1* (*CTR1*) [29], *EIN3-BINDING F BOX PROTEIN 2* (*EBF2*) [30], *ETHYLENE RESPONSE 2* (*ETR2*) [31] etc. However, some well-known ethylene regulated genes, namely *EIN3-BINDING F BOX PROTEIN 1* (*EBF1*) [30], WRKY 25 [32], WRK 26 [32] were not detected by RNA-seq. Thus, ethylene-regulated transcriptome is a good example to exploit the ML-based method to assist the detection of additional genes overlooked in RNA-seq.

Here, by using epigenomics and transcriptomics data from 3-day-old etiolated *Arabidopsis* seedlings of Col-0 and *ein2–*5*,* we tested the performance of ML-based identification of DEGs in response to ethylene. In brief, 468 features were collected from histone H3K9Ac, H3K14Ac and H3K23Ac ChIP-seq data in Col-0 and *ein2–5* mutant seedlings that treated with or without 4 h of ethylene gas. We then identified the top 23 most informative features through assessing the performance of three different feature selection algorithms combined with five different classification methods on training and testing data sets. By comprehensive comparison, we determined that the model based on InfoGain feature selection and Logistic Regression classification is powerful and robust for DEGs prediction. Moreover, the power and performance of ML-based prediction on the expression of ethylene regulated gene were evaluated by qRT-PCR. Taken all together, our study shows that the combination of ML-based method with RNA-seq significantly improved the sensitivity of DEGs identification.

## Methods

### Plant growth conditions

*Arabidopsis* seeds were surface-sterilized in 50% bleach with 0.01% Triton X-100 for 15 min and washed five times with sterile, doubly distilled H_2_O before plating on MS medium (4.3 g MS salt, 10 g sucrose, pH 5.7, 8 g phyto agar per liter). After 3–4 days of cold (4 °C) treatment, the plates were wrapped in foil and kept in at 24 °C in an incubator before the phenotypes of seedlings were analyzed. For propagation, seedlings were transferred from plates to soil (Pro-mix-HP) and grown to maturity at 22 °C under 16-h light/8-h dark cycles. Ethylene treatment of *Arabidopsis* seedlings was performed by growth of seedlings on MS plates in air-tight containers in the dark supplied with either a flow of hydrocarbon-free air (Zero grade air, AirGas) or hydrocarbon-free air with 10 ppm (ppm) ethylene as previously described [33].

### ChIP-seq data analysis

Raw ChIP-seq data associated to histone H3K9Ac, H3K14Ac and H3K23Ac from *Arabidopsis* Col-0 and *ein2–5* etiolated seedlings treated with air or 4 h ethylene gas were downloaded from NCBI GEO under GSE77396 [8] and GSE93875 [34]. The raw reads were mapped to the *Arabidopsis* genome (TAIR10) [35] and uniquely mapped sequencing reads were generated using bowtie software (version 1.1.2) [36, 37]. For each histone modification in each condition, mapped reads were pooled across ChIP-seq replicates as described [8, 34, 38].

### Extraction of segment associated features

**Fig. 1:**
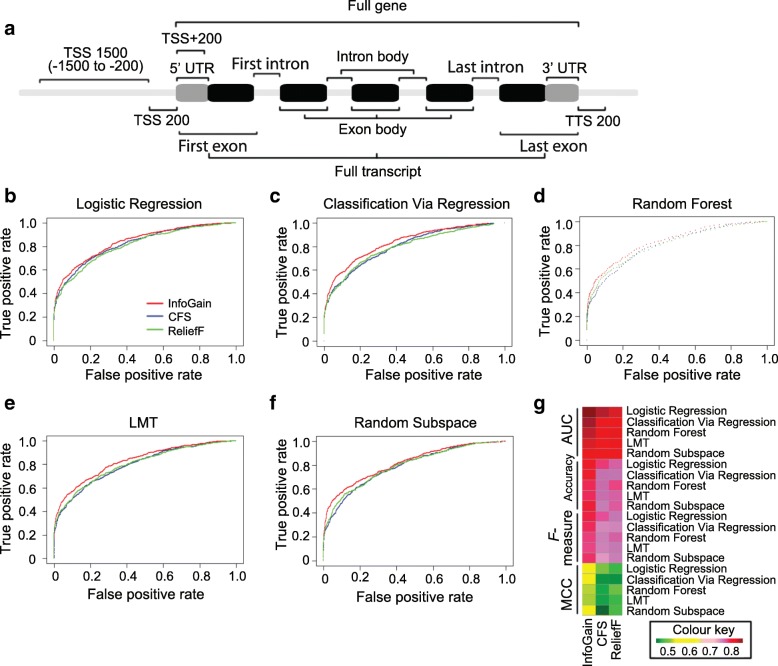
Performance comparison of models with various feature selection and classification methods. **a** Segments associated with protein coding genes. Features considered to predict differential gene expression are depicted on a segment-by-segment basis. From 5′ to 3′ end of the protein coding genes, listed are transcription starting sites (TSS) upstream up to 1500 bp (TSS 1500) and 200 bp (TSS 200), TSS downstream 200 bp (TSS + 200), transcription termination sites (TTS) downstream 200 bp (TTS 200), first exon which may include 5’ UTR, first intron, exon body, last intron, and last exon which may include 3’ UTR. A full transcript region is determined as the UTRs and coding region together. A full gene region is determined as the UTRs, coding region and introns together. **b-f** The Receiver Operating Characteristic (ROC) curves and **g** Areas Under the Curve (AUC) are used to compare the performance of models with different combinations of feature selection (Red line, InfoGain; Blue line, Correlation feature selection (CFS); Green line, ReliefF) and classification (**b** Logistic Regression, **c** Classification Via Regression, **d** Random Forest, **e** Logistic Model Trees (LMT) and **f** Random Subspace), on the training data with 10-fold cross-validation. The model with InfoGain based feature selection and Logistic Regression classification is selected as the best model

The aligned reads were intersected with the relevant segments of the transcript including upstream of the transcription start site (TSS) (TSS1500 and TSS200), downstream of the TSS (TSS + 200), UTR region (UTR5 and UTR3), first and last exon/intron, exon/intron body, single exon/intron, full transcripts and full length of gene (Fig. 1a), using the multicov tool in Bedtools toolkit [39]. These data were further normalized over segment length and total reads number. For the name of features in Additional file 1: Table S2, initials are used to represent the individual ecotype where the features come from: C for the Col-0 seedlings and e for *ein2–5* seedlings. Following the initial is a K started number representing the specific histone H3 acetylation marker: K9 for H3K9Ac, K14 for K3K14Ac, K23 for K3K23Ac. Next to the histone marker is the represent the treatment or comparison of histone modification: A for air treatment, C for ethylene (C_2_H_4_) treatment, diff for the difference of the reads between them were divided by the average of the two [24], and log2FC for log(2)-transformed fold change value between air and ethylene treatment (log2(value of ethylene treatment / value of air treatment)). As a result, features are named as seedling ecotype, histone modification, treatment (or differential histone modification) and segment (such as CK9A_UTR5, CK9diff_UTR5, CK9log2FC_UTR5 etc.).

### Extraction of histone peak associated features

Peaks significantly enriched in ChIP-seq tags were identified by Model-based Analysis for ChIP-Seq (MACS2, version 2.1.0.20150603; parameters: --nomodel,-p 0.01) as previously described [40]. The nearest gene was assigned if there was more than one gene within 5 kilobases (kb) of the peak region [8, 28, 34]. Numbers of peaks assigned to one gene (such as numberpeaks_CK9A), average peak size of peaks assigned to one gene (such as avg_peaksize_CK9A), average peak fold enrichment against IgG control for peaks assigned to one gene (avg_FE_CK9A) and average distance of peaks to the associated gene (such as avg_distance_CK9A) were then calculated.

Differential peaks between air and ethylene treatment were identified using the MAnorm method [41]. For this method, the normalized M value (M = log2 (Read density in C_2_H_4_ treated sample/Read density in air treated sample)) represents log2-transformed fold changes of enrichment intensities at each peak region [40–42]. Thus, an absolute threshold value of M ≥ 0.4 and *P* ≤ 0.05 were used to select differentially enriched peaks as done previously [8, 34]. The nearest gene was assigned if there was more than one gene within 5 kilobases (kb) of the differential peak region [8, 28, 34]. Numbers of differential peaks assigned to one gene (such as numberpeaks_CK9diff), average peak size of differential peaks assigned to one gene (such as avg_peaksize_CK9diff), average peak fold enrichment of ethylene against air treatment for the differential peaks assigned to one gene (avg_FE_CK9diff) and average distance of differential peaks to the associated gene (such as avg_distance_CK9A) were then calculated.

### RNA-seq data analysis

RNA-seq raw data were downloaded from NCBI GEO under GSE77396 [8, 43]. Raw reads were aligned to TAIR10 genome release using TopHat version 2.0.9 [44] with default parameters. Differentially expressed genes were identified using Cufflinks version 2.2.1 following the workflow with default parameters [45]. Gene expression levels (RPKM, Reads Per Kilobase per Million mapped reads) in air and ethylene condition were generated from the output files of cuffdiff [45]. The log2 transformed RPKM values log2 (RPKM) was calculated, and then R scripts were used to analyze the correlation between biological replicates. The differentially expressed genes were then classified as binary outcomes: either up-regulated or down-regulated, once those for which relative fold change values (RPKM) of larger than 1.5 and RPKM value larger than 1 [34].

### Machine learning analysis

Weka 3 data mining software [46] was used for feature selection, classifier training and evaluation. Heat maps, Boxplots, Receiver Operating Characteristic (ROC) curves, Venn diagrams were performed using R (version 3.2.2).

In detail, important features were first identified with three feature selection algorithms: Information Gain (InfoGain) [47]; Correlation Feature Selection (CFS) [24, 47, 48] and ReliefF, [49]. Then, to predict genes in the up and down categories, five widely used classifiers -Logistic Regression, Classification Via Regression, Random Forest, LMT, Random Subspace- were employed, which have been applied to solve various classification and prediction problems in biology, showed comparable or even higher performance than other commonly used machine learning algorithms (Additional file 1: Table S4 and S6) [23, 24, 50–52]. To perform this analysis, we first split the data sets into training data set and testing data set, with 80% of differentially expressed genes for training data set, and the remaining 20% genes for testing data set. Next, to achieve the best combination, 10-fold cross validation on various combinations of feature selection and classification methods were performed. Finally, to predict differentially expressed genes, the top 3 powerful combinations were applied to the candidate gene list. Biological functions of associated genes were assessed by Gene Ontology Consortium [53, 54] and agriGO [55, 56].

### Real-time PCR

Total RNA was extracted using a Qiagen Plant Total RNA Kit (Sigma) from 3-day-etiolated seedlings treated with air or 4 h ethylene gas. First-strand cDNA was synthesized using Invitrogen Superscript III First-Strand cDNA Synthesis Kit. PCR reactions were performed in a total volume of 20 µL, containing 2 µL each 5-mM primer and 10 µL SYBR Green PCR Supermix in triplicate on a Roche 96 Thermal cycler according to the manufacturer’s instruction. The cycling program comprised an initial denaturation step at 95 °C for 10 min, followed by 50 cycles of 95 °C for 10 s, 60 °C for 10 s, and 72 °C for 20 s. All qRT-PCR values were normalized using the cycle threshold value corresponding to the reference gene. The relative expression levels of the target gene were calculated using 2(−Delta Delta C(T)) method [57]. The sequences of all primers are listed in Additional file 1: Table S1.

## Results

### Summary of input data and features

Previous studies revealed that H3K9Ac, H3K14Ac and H3K23Ac were involved in the regulation of gene expression in the response to ethylene [8, 34]. To further assess the connection between these histone modifications and transcriptional regulation systematically, we employed machine learning approach to analyze the features related to the regulation of gene expression. ChIP-seq data of three histone H3 modification markers (H3K9Ac, H3K14Ac and H3K23Ac) and RNA-seq data from Col-0 and *ein2–5* etiolated seedlings treated with or without ethylene gas were used to extract the features. Totally, we collected 468 features which can be divided into two categories (Fig. 1a and Additional file 1: Table S2): (1) histone acetylation over different gene segments, including upstream of the transcription start site (TSS) (TSS1500 and TSS200), UTR region, first and last exon/intron, exon/intron body, single exon/intron, full transcripts and full length of gene. (2) histone peak-associated features, including peak numbers, average peak size, average peak fold enrichment and average distance between peak and the nearest gene.

### Feature selection and evaluation

The Illumina RNA-seq reads files from two biological replicates of Col-0 etiolated seedlings treated with or without ethylene gas were analyzed following the canonical pipeline [58–60] after quality control (Additional file 2: Figure S1). We then split the differentially expressed genes into training and testing data sets, with 2139 genes as training set, and the remaining 535 genes kept as testing set (Additional file 1: Table S3). To identify the most informative features, *F* measure was calculated through Classification Via Regression method [61] in combination with three different feature selection algorithms, including Correlation Feature Selection (CFS) [47, 48], ReliefF [49] and Information Gain (InfoGain) [47], which showed comparable or even better performance than other commonly used machine learning feature selection algorithms (Additional file 1: Table S4). Next, we examined the *F* measure performance when the number of the selected features is the top 1, 2, 3, 4, 5, 6, 7, 8, 9, 10, 20, 30, 40, 50, 60, 70, 80, 90, and 100%, using 10-fold cross-validation on the training data set. Optimal performance was achieved with 4–6% top features used (InfoGain acquires the highest *F* measure 0.774 with 4% top features; CFS acquires the highest *F* measure 0.772 with 5% top features; ReliefF acquires the highest *F* measure 0.768 with 6% top features; Additional file 2: Figure S2). Thus, the top 23 features (top 5%, Additional file 1: Table S5) that further evaluated by five classification methods showed much better performance than other commonly used classification algorithms (Fig. 1, Additional file 1: Table S6). Overall, InfoGain [47] showed a better performance than the other two feature selection algorithms with optimal Receiver Operating Characteristic (ROC) curve and Area Under the ROC Curve (AUC) (Fig. 1 b-g and Additional file 1: Table S4 and S6), the maximum accuracy, and the highest Matthews Correlation Coefficient (MCC) (Fig. 1g and Additional file 1: Table S4). Among the five classification methods examined (Logistic Regression, Classification via Regression, Random Subspace, Random Forest and Logistic model trees (LMT)), Logistic Regression showed the best performances, with an AUC of 0.839 and accuracy of 78.6% for training data (*F* measure = 0.78, MCC = 0.53, Fig. 1 and Additional file 1: Table S4). Taken together, the model based on InfoGain feature selection and Logistic Regression classification was considered the best for the following analysis.

### Analysis of selected features

**Fig. 2:**
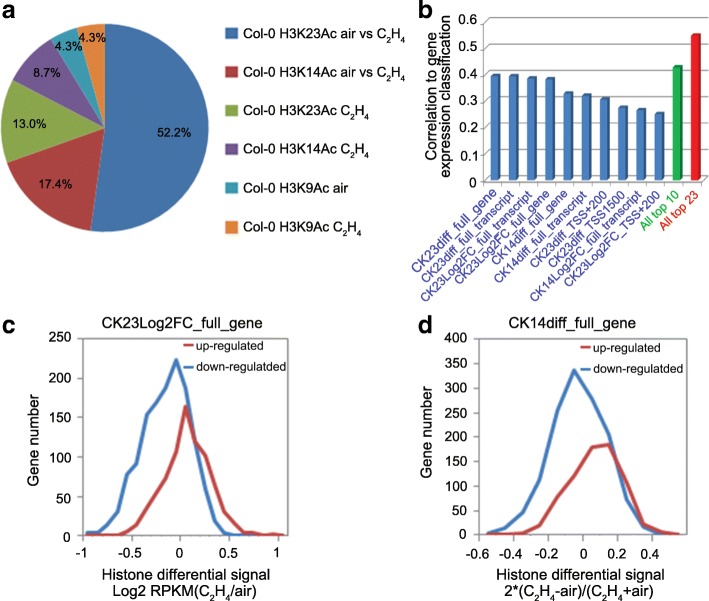
The selected features from the best model. **a** The distribution of different histone markers in the top 23 selected features. **b** List of different features selected by InfoGain feature selection and their correlation to the classification of differential gene expression. The top 10 selected features, which showed in blue, were sorted by their correlation to the differential gene expression. The correlation between all 23 combined features and differential gene expression was showed in red. **c** Distribution of the differential H3K23Ac signals in whole gene regions in Col-0 (CK23Log2FC_full_gene) in ethylene up- and down-regulated genes. **d** Distribution of the differential H3K14Ac signals in whole gene regions in Col-0 (CK14diff_full_gene) in ethylene up- and down- regulated genes

To examine the association of informative features with gene expression regulation in response to ethylene, we analyzed the profile of top 23 features that were selected by InfoGain out of 468 features (Additional file 1: Table S2). Interestingly, all of the selected features were associated with ChIP-Seq data from wild type but not *ein2–*5 mutant, which is completely ethylene insensitive (Fig. 2a and Additional file 1: Table S5), indicating their close relationship to gene expression in response to ethylene. Recent studies have shown a global increase of histone acetylation at H3K14 and H3K23, but not H3K9 in response to ethylene [8]. Consistent with these findings [8], up to 70% of the selected features were associated with differential H3K14Ac and H3K23Ac signals between air and C_2_H_4_ at H3K14 and H3K23 (Fig. 2a and Additional file 1: Table S5). Furthermore, some features associated with H3K14Ac and H3K23Ac signals in gene/transcript with ethylene treatment, or H3K9Ac and H3K23Ac signals in 1500 bp upstream of TSS regions with ethylene treatment, or H3K9Ac signals in 1500 bp upstream of TSS regions without ethylene treatment were selected by InfoGain model (Fig. 2a and Additional file 1: Table S5), indicating the roles of these histone markers in the determination of ethylene regulated genes. Indeed, H3K9Ac before ethylene treatment has been reported to be a potential pre-exist marker for distinguishing up- and down- ethylene regulated genes [34].

Dominant features, if any, would provide insight into the underlying biological process of transcriptional regulation. Hence, we assessed the contribution of top 10 individual features to DEGs identification. Interestingly, none of the top 10 features had a correlation to gene expression score higher than 0.4 (Fig. 2b). We then selected two of top 10 features (CK23_Log2FC_full_gene and CK14diff_full_gene) to compare its distribution in up- and down- regulated genes. The distributions of differential signals of H3K23Ac and H3K14Ac were distinct in ethylene up-regulated gene from ethylene down-regulated genes. However, a significant overlap was also detected (Fig. 2c and d), suggesting that a single feature is not sufficient to predict the gene expression. However, the correlation increased with 23 features incorporated into the model (Fig. 2b). This additive effect of combined features in classification indicated unrevealed interplay among epigenetic markers, which in turn addressed the usefulness of machine learning in such complicated biological contexts.

### Comparison of prediction using different models

**Fig. 3:**
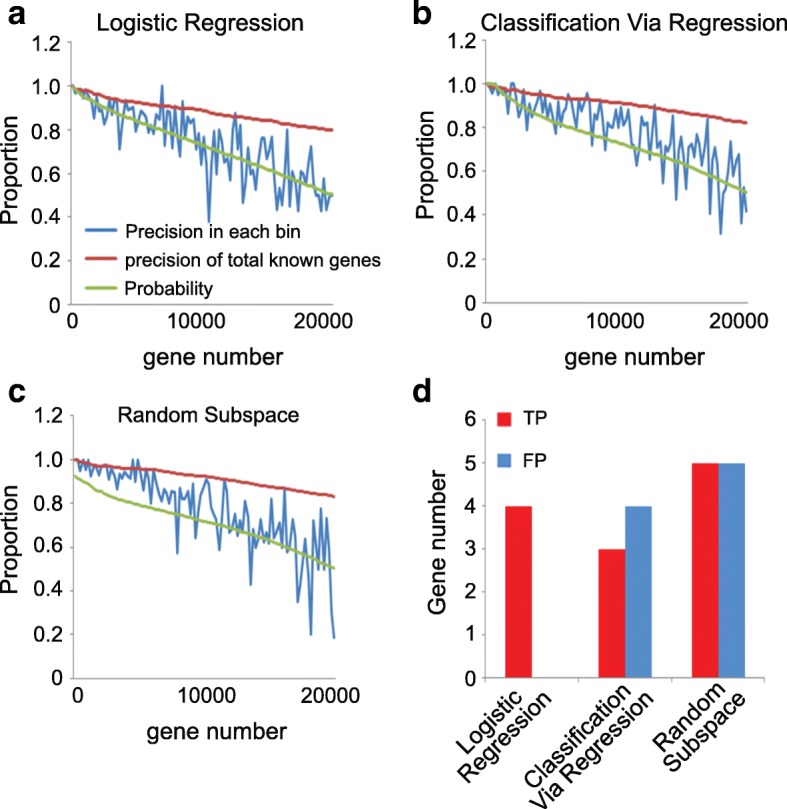
Comparative Evaluation different machine learning based models. **a-c** For each predicted gene list, the class probability estimation (green line), the predicted precision of true positive genes in each bin (blue line) and the predicted precision of total known predicted genes (red line) were plotted to illustrate the prediction accuracy of **a** Logistic Regression, **b** Classification Via Regression and **c** Random Subspace based methods. **d** Number of true positive (TP) or false positive (FP) genes that predicted by different methods using ChIP-Seq data from *ein2–5*

Next, we compare the performance of the models that were defined as the top 3 powerful ones, that are the model based on InfoGain feature selection and Logistic Regression classification, Classification Via Regression and Random Subspace classification for genes prediction (Additional file 1: Table S4) by using the high or medium (top 60%) expressed genes, including most of ethylene regulated genes (97.8%) [34]. The genes predicted by each classifier were ranked by class probability estimation from high to low, and were then grouped with 200 genes per bin. Furthermore, the predicted precision of true positive genes in known ethylene regulated genes in each bin was calculated. The precision decreased with the decreasing of probability estimation for the genes predicated by each classifier (Fig. 3a-c), showing a good performance of data processing. We then calculated the total predicted genes with a standard that total precision larger than 0.95 with predicted candidate genes. Finally, 2600, 4600 and 6400 genes were predicted by the above, respectively (Additional file 1: Table S7). Majority of predicated genes by the model based on InfoGain feature selection and Logistic Regression classification were overlapped with that predicated by Classification Via Regression and Random Subspace (Additional file 2: Figure S3a).

To further examine the performance of selected model, we used 23 features in Col-0 to test the prediction of gene expression in *ein2–5* mutant, which is ethylene insensitive. To ensure a more accurate assessment, we only used differentially expressed genes with absolute fold change larger than 4 in *ein2–5* for further analysis. Moreover, the same number of predicted genes with Col-0 was selected for further analysis. All the predicted genes by the model based on InfoGain and Logistic Regression showed the same regulation by ethylene as the result from RNA-seq (Referred to as true positive genes, TP, Fig. 3d), and 4 of them are known differentially expressed genes in *ein2–5* [62]. In contrast, by using Classification Via Regression and Random Subspace methods, almost half of the known predicted genes failed to match RNA-seq results (Referred to as false positive genes, FP, Fig. 3d). To evaluate whether the number of predicted genes selected affects the accuracy of prediction, we compared the performance of these three methods when the same number of predicted genes were selected. We found that the model based on InfoGain and Logistic Regression was the most powerful one to achieve the largest numbers of TP genes and lowest numbers of FP (Additional file 2: Figure S3b). Taken together, these results indicate that the model based on InfoGain and Logistic Regression has the best performance on DEGs prediction, and these predicted genes are highly likely differentially regulated by ethylene in etiolated *Arabidopsis* seedlings.

### Analysis and validation of newly predicted genes

**Fig. 4:**
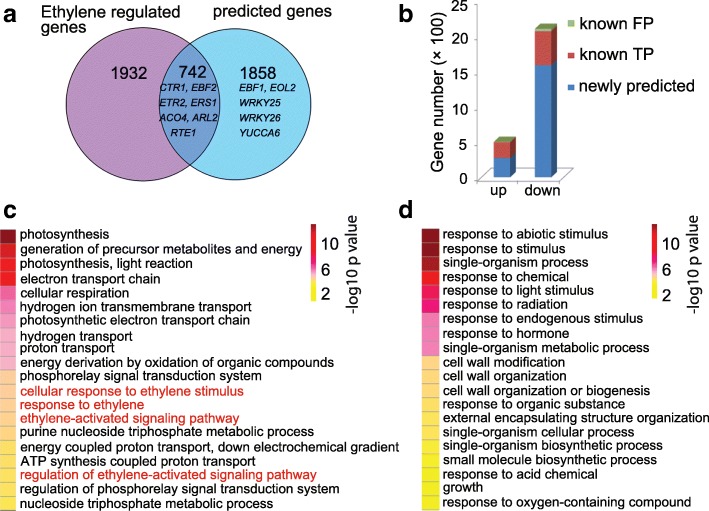
Analysis of ethylene regulated genes predicted by the model based on InfoGain feature selection and Logistic Regression classification. **a** Almost one third of predicted genes are known ethylene regulated genes. **b** A statistical analysis of known ethylene regulated genes that show true positive (known TP) and false positive (known FP) genes in up and down predicated genes. **c** GO analysis of top 20 biological processes enriched in the up regulated genes that predicated by the best model. **d** GO analysis of top 20 biological processes enriched in the down regulated genes that predicated by the best model

Among 2600 genes predicted by InfoGain and Logistic Regression in Col-0, 742 genes were well-known ethylene regulated, including *CONSTITUTIVE TRIPLE RESPONSE 1* (*CTR1*) [29], *EIN3-BINDING F BOX PROTEIN 2* (*EBF2*) [30], and *ETHYLENE RESPONSE 2* (*ETR2*) [31] (Fig. 4a, p-value = 3.97E-116, Hypergeometric Distribution test), thus confirmed the reliability of our method. Remarkably, among the 1858 newly predicted genes, *EIN3-BINDING F BOX PROTEIN 1* (*EBF1*) [30], *WRKY 25* [32], *WRKY 26* [32] and *YUCCA6* [63] that were missed by RNA-seq were presented, showing the power of our model in prediction. Consistent with previous reports, more down-regulated genes were predicted than up-regulated ones (Fig. 4b). Nonetheless, there is still room for improvement in accuracy of our model, as the predicted down-regulated *WEAK ETHYLENE INSENSITIVE 8* (*WEI8/CKRC1*) has been reported to be induced by ethylene [64].

Gene ontology (GO) enrichment analysis revealed that the group of predicted up-regulated genes was enriched with genes involved in response to ethylene (Fig. 4c and Additional file 1: Table S8). And the predicted down-regulated genes were more involved in response to abiotic and endogenous/hormone stimulus, cell wall modification, and basic metabolic/biosynthetic processes (Fig. 4d and Additional file 1: Table S8). Further GO enrichment analysis showed that ethylene-associated genes were overrepresented in those genes broadly annotated as “response to hormone” in predicted down-regulated genes (Additional file 1: Table S8).

**Fig. 5:**
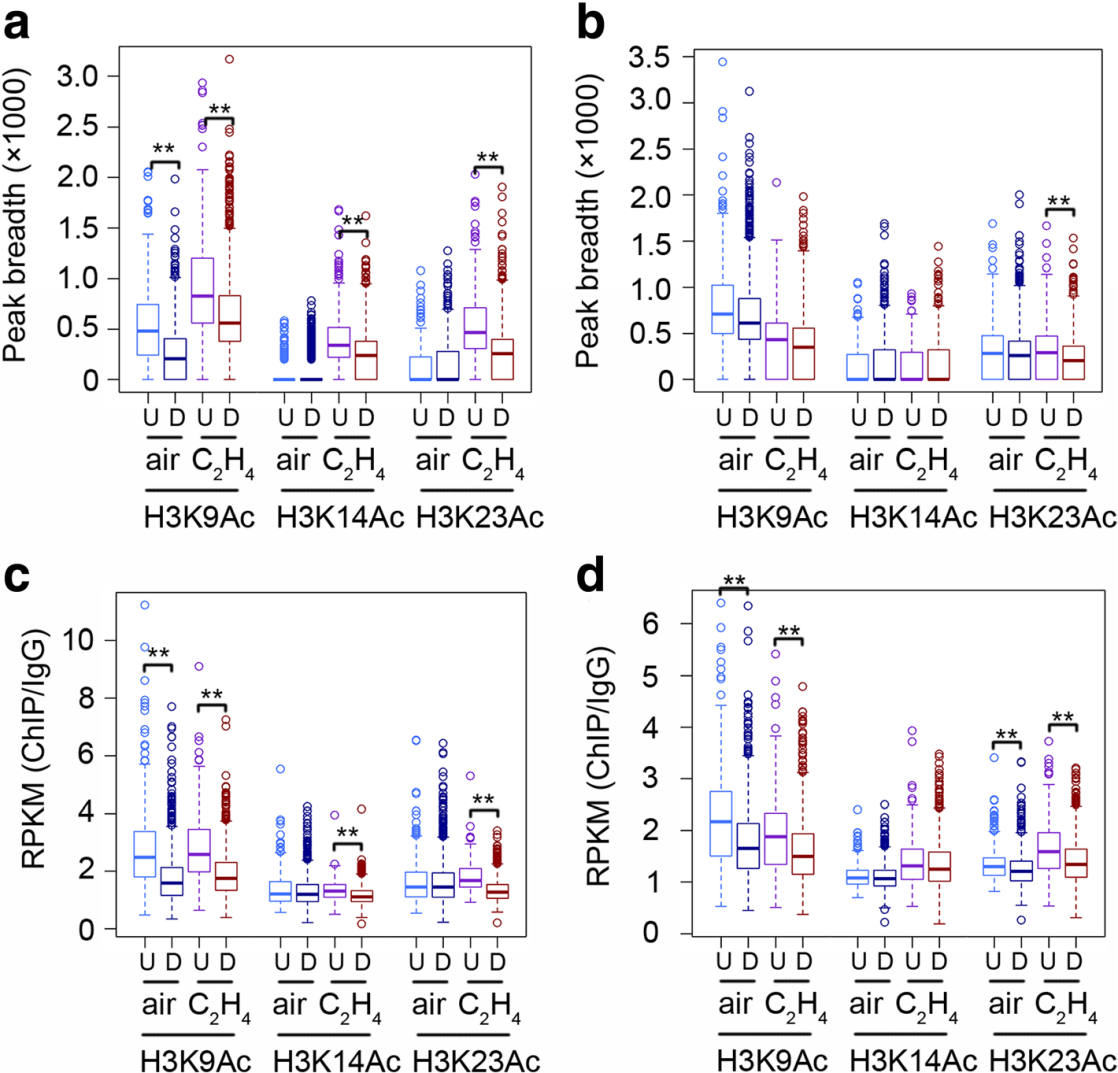
Histone acetylation patterns in predicted genes. **a** and **b** Boxplot showing the correlation of peak breadths and ethylene up-regulated genes (U, *n* = 360) or ethylene down-regulated genes (D, *n* = 1508) in **a** Col-0 or **b** *ein2–5* under air and ethylene treatment. The ** indicates *P* < 0.001 by t-test. **c** and **d** Boxplot showing the histone mark enrichment (RPKM) in 1000 bp around TSSs in ethylene up-regulated genes (U, n = 360) and ethylene down-regulated genes (D, n = 1508) in **c** Col-0 or **d** *ein2–5* in air and ethylene. The ** indicates P < 0.001 by t-test

We next divided predicted ethylene-regulated genes marked by H3K9Ac, H3K14Ac and/or H3K23Ac into two groups as ethyleneup-regulated genes (*n* = 360) and ethylene down-regulated genes (*n* = 1508), and analyzed the signals of H3K9Ac, H3K14Ac and H3K23Ac associated with them. In the absence of ethylene, peak breadths of H3K9Ac were larger in the predicted ethylene up-regulated genes than in down-regulated genes, but no difference were detected for H3K14Ac and H3K23Ac (Fig. 5a). In the presence of ethylene, the peak breadths for each of the three histone marks in predicted up- and down- regulated genes were elevated, and the peak breadths became larger in up-regulated genes than that in down-regulated genes (Fig. 5a). Interestingly, all these ethylene-induced changes in Col-0 were not detected (or strongly decreased in H3K23Ac) in *ein2–5* mutant (Fig. 5b). Although none of the peak breadth-related features were used for the prediction, these results were consistent with the peak breadth distribution of known ethylene regulated genes except the H3K9Ac in the presence of ethylene [34].

**Fig. 6:**
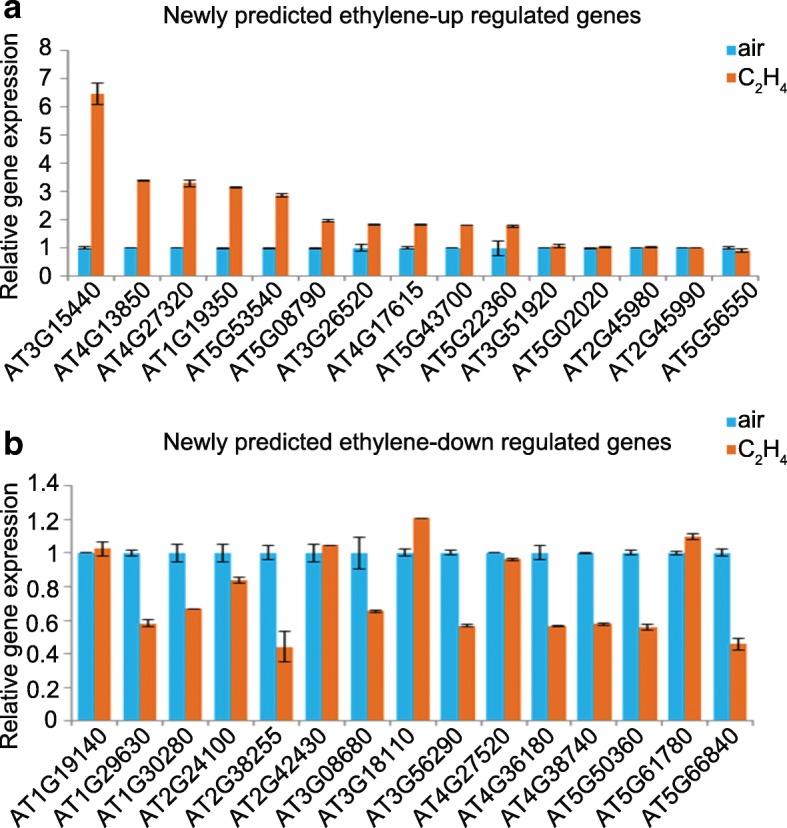
qRT-PCR validation of genes randomly selected from predicted genes. **a** 15 newly predicated ethylene up-regulated genes were randomly selected for qRT-PCR test using 3-day-old etiolated seedlings treated with air or 4 h of ethylene gas. **b** 15 newly predicated ethylene up-regulated genes were randomly selected for qRT-PCR test using 3-day-old etiolated seedlings treated with air or 4 h of ethylene gas. Data represent the relative fold change. Each experiment has two biology replicates with similar result

In the presence of ethylene, the H3K9Ac signal was not altered in those predicted genes (Fig. 5c), which was similar as in those known ethylene-regulated genes reported previously [8], whereas, the levels of H3K9Ac were higher in the ethylene up-regulated genes than that in the down-regulated genes. In contrast to H3K9Ac, the levels of H3K14Ac and H3K23Ac over the predicted genes showed a positive association with the gene expression in the response to ethylene (Fig. 5c). Interestingly, the predicted ethylene-induced alterations in gene expression in Col-0 were reduced or not detected in *ein2–5* mutant (Fig. 5d). Taken together, these results suggest that the prediction of changes in gene expression conducted by our model based on InfoGain and Logistic Regression achieved an impressive level of accuracy. To confirm the accuracy of the ML-based gene prediction, we randomly selected 15 predicted up and down genes for qRT-PCR assay. More than 60% of the selected genes behaved consistently between the prediction and qRT-PCR validation (Fig. 6a and b).

### ML based methods application on other organisms

To test the performance of ML based methods on other organisms, we downloaded data from the ENCODE Consortium (http://genome.ucsc.edu/ENCODE/downloads.html) for two human cell lines: lymphoblastoid cells (Gm12878) and Hela S3 cells. We collected genome-wide histone modification for H3K4me3, H3K9Ac and H3K27me3, as well as differentially expressed genes from RNA-seq data between these two cell lines. Totally, we collected 114 features which included histone modification on different gene segments and histone peak-associated features, as mentioned before. After *F* measure evaluation for three feature selection algorithms (InfoGain, CFS and ReliefF), the top 46 features (top 40%, Additional file 2: Figure S4a) were further studied by five classification methods. Overall, the model based on InfoGain feature selection and RandomForest classification was considered the best model for the following analysis (AUC = 0.996 for training data sets, Additional file 2: Figure S4b), and the top 60% highly expressed genes were used for prediction (Additional file 2: Figure S4c). Considering the better performance of ML based method on human cells than *Arabidopsis* (such as better AUC on training data and predicted data), the precision greater than 0.9995 was used to select predicted candidate genes (Additional file 2: Figure S4d and S4e). Among 13,200 predicted candidate genes, 5260 (~ 40%) genes are known DEGs (Additional file 2: Figure S4f), and only two false positive genes were predicted. GO terms from predicted candidate genes were enriched in the regulation of cell proliferation and developmental process, biological functions related pathways, which is consistent with a previous study [65]. Overall, the result shows that ML based method is also applicable in human cells.

To further validate our approach, we downloaded data from Gene Expression Omnibus database (accession number GSE68299) for two rice samples [66]: wild type (*Oryza sativa*) and SET DOMAIN GROUP 711 RNAi plants (SDG711RNAi). We collected genome-wide histone modifications for H3K4me3 and H3K27me3, and differentially expressed genes from RNA-seq data between these two rice lines. Totally, 84 features were collected and the top 25 features (top 30%, Additional file 2: Figure S5a) were further studied by five classification methods. Overall, the model based on InfoGain feature selection and RandomForest classification was considered as the best model for the following analysis (AUC = 0.718 for training data sets, Additional file 2: Figure S5b). We used top 40% highly expressed genes for prediction (Additional file 2: Figure S4c) and the precision greater than 0.99 was used to select the predicted candidate genes (Additional file 2: Figure S5d). Among 5600 predicted candidate genes, 831 (~ 15%) genes are known DEGs (Additional file 2: Figure S5e), in which only has 8 false positive genes were predicted. Consistent with the function of SDG711 that affect panicle size [66], many development and cell growth/size related genes were enriched in GO analysis (Additional file 2: Figure S5f).

## Discussions

Although remarkable power of RNA-seq has been achieved in the past few years, currently available methods leave rooms for improvement in terms of sensitivity and efficiency that are greatly affected by experimental design/operation and the following data analysis processes [9, 67, 68]. To fill in this gap, two strategies have been employed: to improve experimental design/performance and to develop better algorithms/softwares [13, 14, 16, 67, 68]. Machine learning based methods provide a new way that could avoid the inherited limitations existed in experimental design or data analysis processes in RNA-seq. It has been used to predict stress-related genes in *Arabidopsis* [23] or cancer related genes in human [24]. In this study, we found that more than 70% of the predicted genes were never before reported as DEGs in the response to ethylene, thus provided us a greatly enlarged candidate pool for future research and showed the power of machine learning based method in the predcition of  novel DEGs.

To validate our prediction, we examined the expression of the newly identified genes by qRT-PCR. Up to 60% of newly predicted genes were confirmed to be regulated by ethylene (Fig. 6). Yet, the accuracy of machine learning based method still needs improvement. Given the close relationship among gene expression, histone methylation [69], histone acetylation [70], DNA methylation [71] RNA methylation [72], and post transcriptional regulation, further studies including additional features such as other histone modifications, DNA modification, RNA modification or post transcriptional regulation would help improve the accuracy of prediction. In addition, we were not able to estimate gene expression quantitatively based on binary classification methods, which could be realized by emerging advanced models in deep learning, such as MultiLayer Perceptron and Stacked Denoising Auto-encoder [73].

The result in our study indicates that the 23 features including all H3K9Ac, K14Ac and K23Ac data were selected by InfoGain feature selection method contribute to the gene expression regulation in the response to ethylene (Fig. 2a). Differential acetylation levels of H3K14 and H3K23 are the largest two groups among selected features by InfoGain and Logistic Regression. In addition, the level of H3K9Ac in air condition was selected as an important feature as well. Indeed, recent studies have shown that the global increase in histone H3K14Ac and H3K23Ac was tightly associated with ethylene-regulated gene expression [8], whereas the levels of H3K9Ac were not regulated by ethylene. However, the levels of H3K9Ac in the ethylene up-regulated genes were significantly higher than that in the ethylene down-regulated genes. Therefore the H3K9Ac levels are considered as a potential pre-exist marker for distinguishing up- and down- regulated genes in the ethylene response [34]. It is well known that the promoter regions are critical for transcriptional regulation, histone modification in the promoter regions is one of the most important features for the prediction of gene expression. Our results obtained by using the model based on InfoGain and Logistic Regression demonstrate that the genomic locations that relative to each transcript including promoters, exons and gene bodies (Additional file 1: Table S5) can provide useful information for the prediction of gene expression.

## Conclusion

RNA-seq is a widely used technique for transcriptome profiling, but there are still inherited limitations on the detectability for certain DEGs due to the limitations in experimental or data analysis processes. By comprehensive comparison, we determined that the model based on InfoGain feature selection and Logistic Regression classification is powerful and robust for DEGs prediction. Moreover, the power and performance of ML-based prediction on ethylene regulated gene expression were evaluated by qRT-PCR. Taken all together, our study shows that the combination of ML-based method with RNA-seq data analysis significantly improved the sensitivity of the DEGs identification. Further studies should be conducted to improve performance of ML based methods by using more epigenomics data and advanced models in deep learning.

## Additional files

Additional file 1: **Table S1.** Primers for qPCR. **Table S2.** The list of all features considered prior to feature selection. **Table S3.** Training and testing data set used for machine learning. **Table S4.** Summary of feature evaluation. **Table S5.** Selected features via differential models. **Table S6.** AUC values of different feature selection methods with combination of different classification methods. **Table S7.** Predicted genes by the model based on InfoGain feature selection and Logistic Regression. **Table S8.** GO analysis of predicted genes. (ZIP 215 kb)

Additional file 2: **Figure S1.** RNA-seq quality detection of differential regulated genes in air and ethylene treatment. **Figure S2.** The *F* measure performance curves of all the feature selection methods on the training data when the number of the selected features is the top 1%, 2%, 3%, 4%, 5%, 6%, 7%, 8%, 9%, 10%, 20%, 30%, 40%, 50%, 60%, 70%, 80%, 90%, and 100%. **Figure S3.** Evaluation of predicted genes. **Figure S4.** Evaluation in human cells**. Figure S5.** Evaluation in rice. (PDF 1302 kb)

## Abbreviations

AUC: Area Under the ROC Curve
CFS: Correlation Feature Selection
ChIP-seq: Chromatin immunoprecipitation sequencing
CTR1: CONSTITUTIVE TRIPLE RESPONSE 1
DEGs: Differentially expressed genes
EBF1: EIN3-BINDING F BOX PROTEIN 1
EBF2: EIN3-BINDING F BOX PROTEIN 2
ETR2: ETHYLENE RESPONSE 2
GO: Gene ontology
InfoGain: Information Gain
LMT: Logistic model trees
MACS2: Model-based Analysis for ChIP-Seq version 2.1.0.20150603
MCC: Matthews Correlation Coefficient
ML: Machine learning
qRT-PCR: Real-time reverse transcription PCR
RNA-seq: RNA sequencing
ROC: Receiver Operating Characteristic
RPKM: Reads Per Kilobase per Million mapped reads
WEI8: WEAK ETHYLENE INSENSITIVE 8
