# Supplementary material for: RNA-seq assistant: machine learning based methods to identify more transcriptional regulated genes
Source: BMC Genomics. 2018 Jul 20;19:546. doi: 10.1186/s12864-018-4932-2 (PMC6053725; doi:10.1186/s12864-018-4932-2)
Supplement: Supplementary file 2 — Figure S1. RNA-seq quality detection of differential regulated genes in air and ethylene treatment. Figure S2. The F measure performance curves of all the feature selection methods on the training data when the number of the selected features is the top 1%, 2%, 3%, 4%, 5%, 6%, 7%, 8%, 9%, 10%, 20%, 30%, 40%, 50%, 60%, 70%, 80%, 90%, and 100%. Figure S3. Evaluation of predicted genes. Figure S4. Evaluation in human cells. Figure S5. Evaluation in rice. (PDF 1302 kb) [file 12864_2018_4932_MOESM2_ESM.pdf]

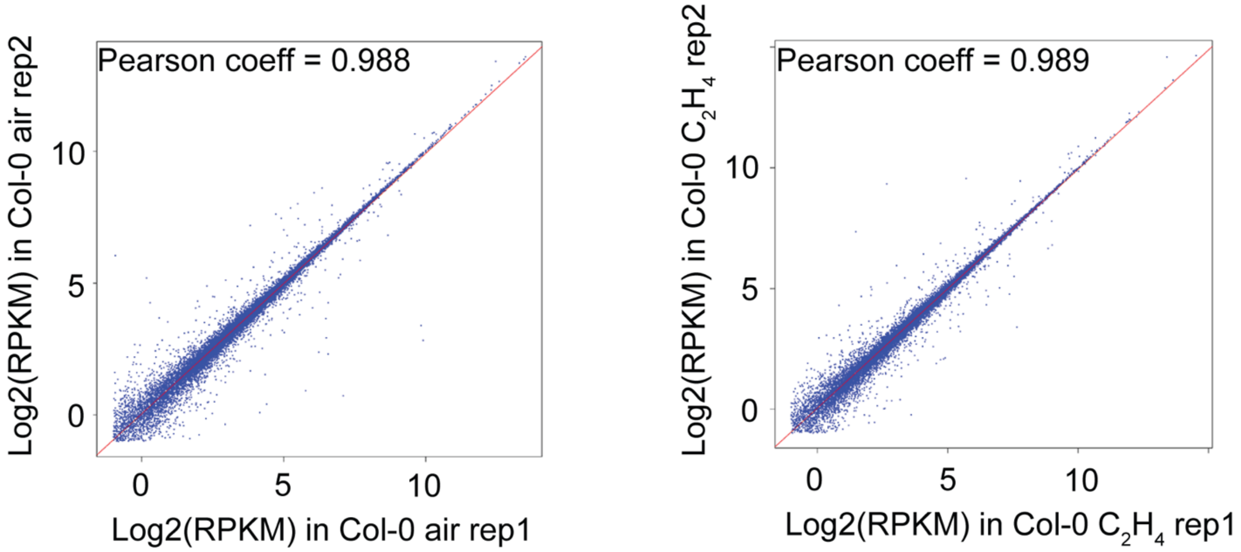

Additional file 2: Figure S1. RNA-seq quality detection of differential regulated genes in air and ethylene treatment.

Scatter plots of gene expression level show quality of RNA-seq data in Col-0 treated with air (left panel) and ethylene gas (right panel).

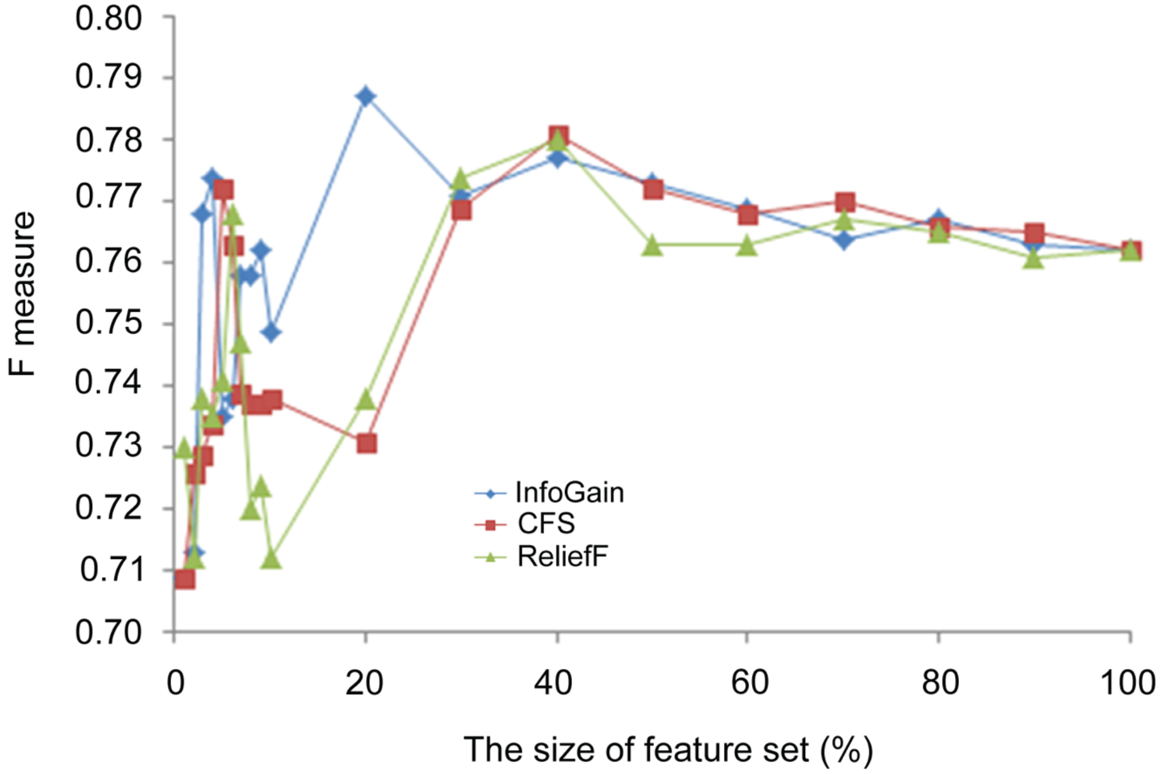

Additional file 2: Figure S2. The *F* measure performance curves of all the feature selection methods on the training data when the number of the selected features is the top 1%, 2%, 3%, 4%, 5%, 6%, 7%, 8%, 9%, 10%, 20%, 30%, 40%, 50%, 60%, 70%, 80%, 90%, and 100%.

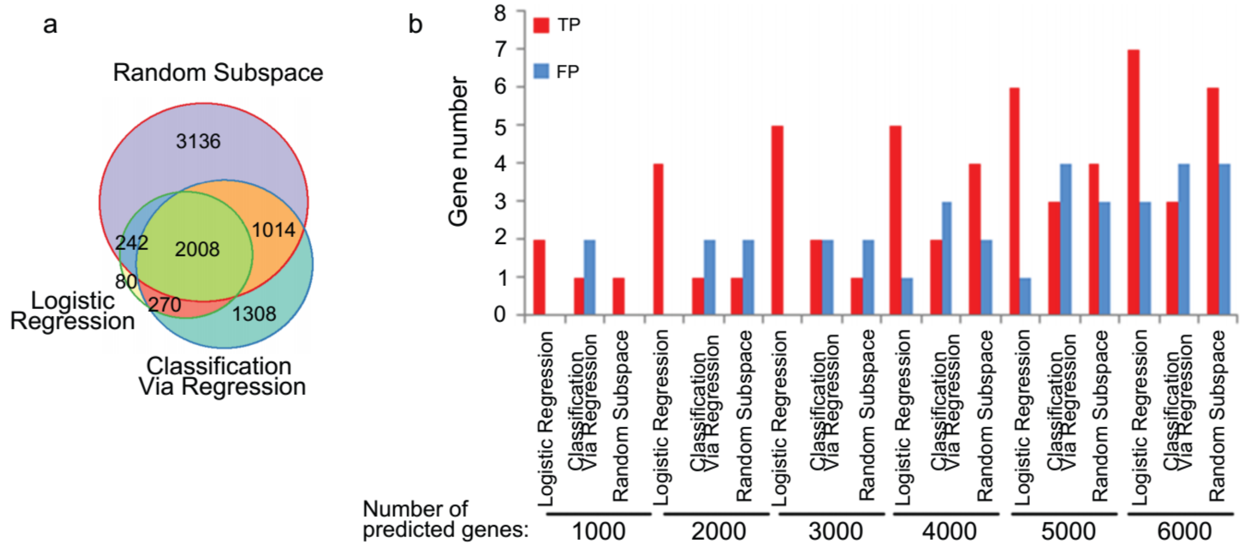

Additional file 2: Figure S3. Evaluation of predicted genes.

a. overlap of predicted genes that produced by Random Subspace, Logistic Regression and Classification Via Regression methods.

b. Evaluation of performance of selected model on gene prediction in ein2-5 mutant. The true positive and false positive of known ethylene regulated genes in ein2-5 were calculated when the total candidate genes is the top 1000, 2000, 3000, 4000, 5000, and 6000.

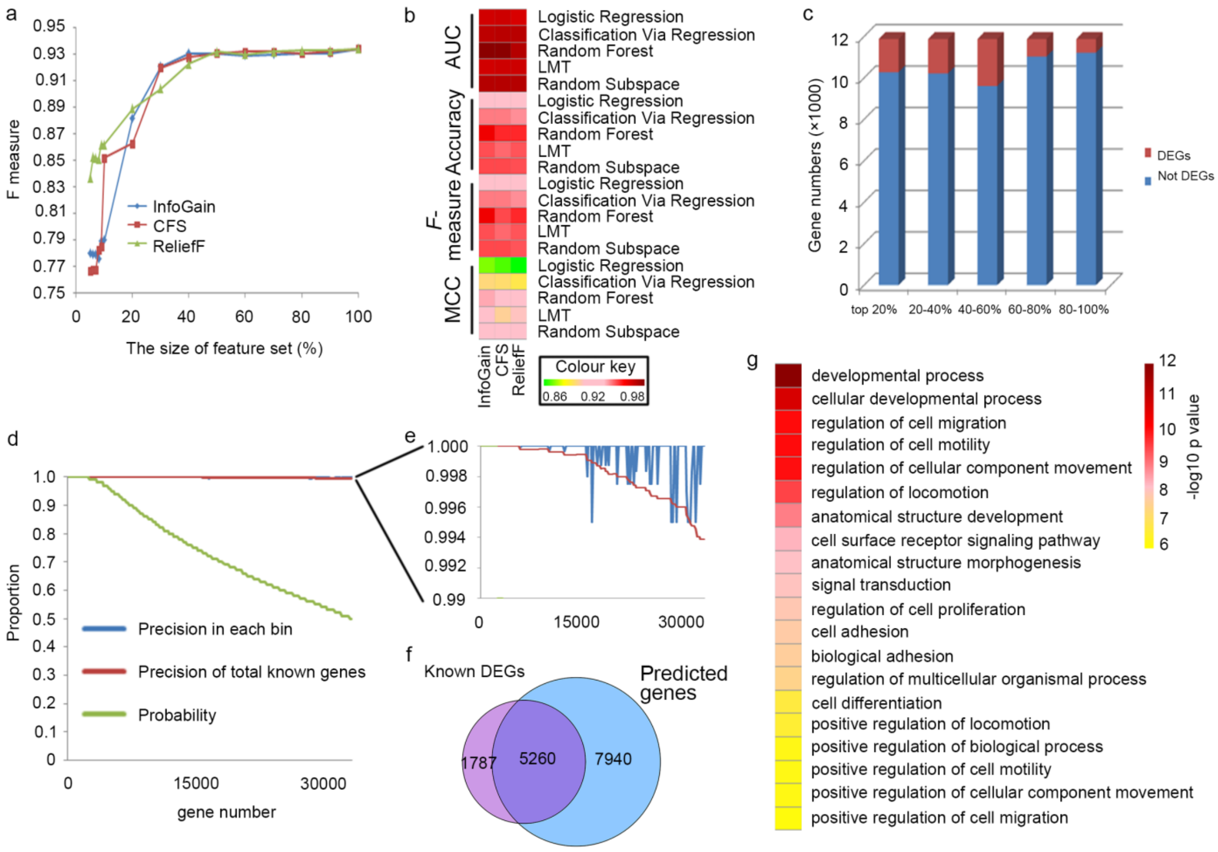

Additional file 2: Figure S4. Evaluation in human cells

a. The *F* measure performance curves of all the feature selection methods on the training data when the number of the selected features is the top 5%, 6%, 7%, 8%, 9%, 10%, 20%, 30%, 40%, 50%, 60%, 70%, 80%, 90%, and 100%.

b. The Receiver Operating Characteristic (ROC) curves and Areas Under the Curve (AUC) were used to compare the performance of models with different combinations of feature selection and classification, on the training data with 10-fold cross-validation. The model with InfoGain based feature selection and RandomForest was selected as the best model.

c. Numbers of human genes that are differentially expressed and not differentially expressed between Gm12878 and Hela S3 cell lines based on relative mRNA expression levels were divided into five equal sets.

d. For predicted gene list, the class probability estimation (green line), the predicted precision of true positive genes in each bin (blue line) and the predicted precision of total known predicted genes (red line) were plotted to illustrate the prediction accuracy of RandomForest based methods.

e. An enlargement of the regions from 0.99 to 1 of figure **d**.

f. Almost 40% of predicted genes are known ethylene regulated genes. Only 2 genes were predicted as false positive among 5260 known DEGs.

g. GO analysis of top 20 biological processes enriched in the genes that predicated by the best model.

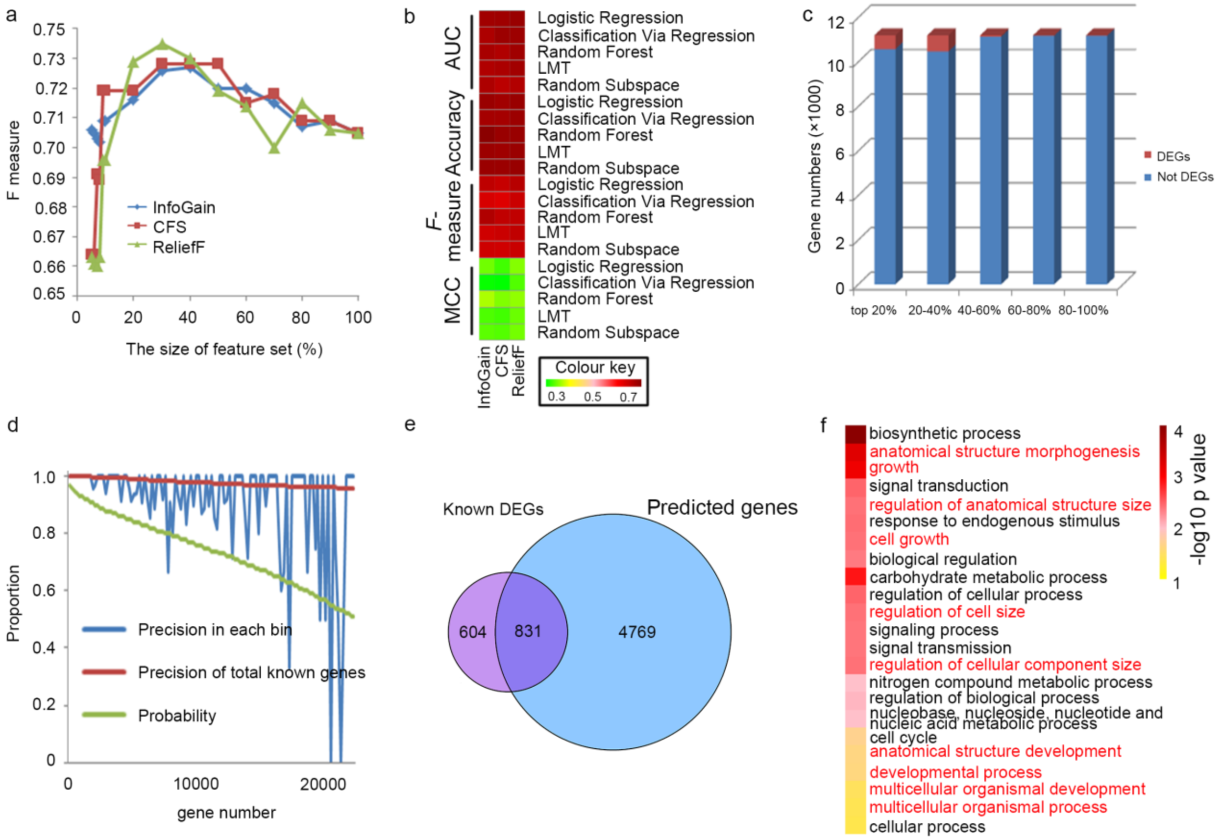

Additional file 2: Figure S5. Evaluation in rice

a. The *F* measure performance curves of all the feature selection methods on the training data when the number of the selected features is the top 5%, 6%, 7%, 8%, 9%, 10%, 20%, 30%, 40%, 50%, 60%, 70%, 80%, 90%, and 100%.

b. The Receiver Operating Characteristic (ROC) curves and Areas Under the Curve (AUC) are used to compare the performance of models with different combinations of feature selection and classification, on the training data with 10-fold cross-validation. The model with InfoGain based feature selection and RandomForest is selected as the best model.

c. Numbers of rice genes that are differentially expressed and not differentially expressed between SDG711RNAi and wild type rice based on relative mRNA expression levels divided into five equal sets.

d. For predicted gene list, the class probability estimation (green line), the predicted precision of true positive genes in each bin (blue line) and the predicted precision of total known predicted genes (red line) were plotted to illustrate the prediction accuracy of RandomForest based methods.

e. Almost 15% of predicted genes are known ethylene regulated genes. 8 genes were predicted as false positive among 831 known DEGs.

f. GO analysis of biological processes enriched in the genes that predicated by the best model.
